# A genome-wide association study on meat consumption in a Japanese population: the Japan Multi-Institutional Collaborative Cohort study

**DOI:** 10.1017/jns.2021.49

**Published:** 2021-10-11

**Authors:** Yasuyuki Nakamura, Akira Narita, Yoichi Sutoh, Nahomi Imaeda, Chiho Goto, Kenji Matsui, Naoyuki Takashima, Aya Kadota, Katsuyuki Miura, Masahiro Nakatochi, Takashi Tamura, Asahi Hishida, Ryoko Nakashima, Hiroaki Ikezaki, Megumi Hara, Yuichiro Nishida, Toshiro Takezaki, Rie Ibusuki, Isao Oze, Hidemi Ito, Nagato Kuriyama, Etsuko Ozaki, Haruo Mikami, Miho Kusakabe, Hiroko Nakagawa-Senda, Sadao Suzuki, Sakurako Katsuura-Kamano, Kokichi Arisawa, Kiyonori Kuriki, Yukihide Momozawa, Michiaki Kubo, Kenji Takeuchi, Yoshikuni Kita, Kenji Wakai

**Affiliations:** 1 Department of Public Health, Shiga University of Medical Science, Otsu, Japan; 2 Yamashina Racto Clinic and Medical Examination Center, Kyoto, Japan; 3 Department of Integrative Genomics, Tohoku Medical Megabank Organization, Tohoku University, Sendai, Japan; 4 Division of Biomedical Information Analysis, Institute for Biomedical Sciences, Iwate Medical University, Shiwa-gun, Iwate, Japan; 5 Department of Nutrition, Faculty of Wellness, Shigakkan University, Obu, Japan; 6 Department of Public Health, Nagoya City University Graduate School of Medical Sciences, Nagoya, Japan; 7 Department of Health and Nutrition, School of Health and Human Life, Nagoya Bunri University, Inazawa, Japan; 8 Division of Bioethics and Healthcare Law, The National Cancer Center, Tokyo, Japan; 9 Department of Public Health, Faculty of Medicine, Kindai University, Osaka-Sayama, Osaka, Japan; 10 NCD Epidemiology Center, Shiga University of Medical Science, Otsu, Japan; 11 Public Health Informatics Unit, Department of Integrated Health Sciences, Nagoya University Graduate School of Medicine, Nagoya, Japan; 12 Department of Preventive Medicine, Nagoya University Graduate School of Medicine, Nagoya, Japan; 13 Department of General Internal Medicine, Kyushu University Hospital, Fukuoka, Japan; 14 Department of Comprehensive General Internal Medicine, Faculty of Medical Sciences, Kyushu University Graduate School, Fukuoka, Japan; 15 Department of Preventive Medicine, Faculty of Medicine, Saga University, Saga, Japan; 16 Department of International Island and Community Medicine, Kagoshima University Graduate School of Medical and Dental Sciences, Kagoshima, Japan; 17 Division of Cancer Epidemiology and Prevention, Aichi Cancer Center, Nagoya, Japan; 18 Division of Cancer Information and Control, Aichi Cancer Center, Nagoya, Japan; 19 Division of Descriptive Cancer Epidemiology, Nagoya University Graduate School of Medicine, Nagoya, Japan; 20 Department of Epidemiology for Community Health and Medicine, Kyoto Prefectural University of Medicine, Kyoto, Japan; 21 Department of Social Health Medicine, Shizuoka Graduate University of Public Health, Shizuoka, Japan; 22 Cancer Prevention Center, Chiba Cancer Center Research Institute, Chiba, Japan; 23 Department of Preventive Medicine, Tokushima University Graduate School of Biomedical Sciences, Tokushima, Japan; 24 Laboratory of Public Health, Division of Nutritional Sciences, School of Food and Nutritional Sciences, University of Shizuoka, Shizuoka, Japan; 25 Laboratory for Genotyping Development, RIKEN Center for Integrative Medical Sciences, Kanagawa, Japan; 26 Faculty of Nursing Science, Tsuruga Nursing University, Tsuruga, Japan

**Keywords:** Genome-wide association study, Meat consumption, Rs671, *ALDH2*, aldehyde dehydrogenase 2, BMI, body mass index, FFQ, food frequency questionnaire, GWAS, genome-wide association study, J-MICC, Japan Multi-Institutional Collaborative Cohort, PCA, principal component analysis, Q–Q, quantile–quantile, SNP, single nucleotide polymorphism

## Abstract

Recent genome-wide association studies (GWAS) on the dietary habits of the Japanese population have shown that an effect rs671 allele was inversely associated with fish consumption, whereas it was directly associated with coffee consumption. Although meat is a major source of protein and fat in the diet, whether genetic factors that influence meat-eating habits in healthy populations are unknown. This study aimed to conduct a GWAS to find genetic variations that affect meat consumption in a Japanese population. We analysed GWAS data using 14 076 participants from the Japan Multi-Institutional Collaborative Cohort (J-MICC) study. We used a semi-quantitative food frequency questionnaire to estimate food intake that was validated previously. Association of the imputed variants with total meat consumption per 1000 kcal energy was performed by linear regression analysis with adjustments for age, sex, and principal component analysis components 1–10. We found that no genetic variant, including rs671, was associated with meat consumption. The previously reported single nucleotide polymorphisms that were associated with meat consumption in samples of European ancestry could not be replicated in our J-MICC data. In conclusion, significant genetic factors that affect meat consumption were not observed in a Japanese population.

## Introduction

A wealth of information shows that higher consumption of meat, especially red meat and processed red meat, is associated with a higher risk of type 2 diabetes, cardiovascular disease, certain types of cancers and all-cause mortality^(1–6)^. Despite this, meat consumption in Japan is increasing due to the westernisation of the diet^(6)^. However, meat consumption in Japan is still less than half of that in the USA^(7)^. In a region where meat consumption is in a lower range, some beneficial effects of meat consumption on health outcomes have been observed. That is, a recent cohort study by Saito *et al.* showed that total meat intake was associated with a lower risk of stroke mortality in women, although heavy intake of total meat, and red meat was associated with an increase in all-cause and heart disease mortality in men^(6)^.

Differences in some individual eating habits are influenced by genetic factors, in addition to cultural, social or environmental factors. For instance, researches with the candidate gene approach suggested that genetic variants of the sweet taste receptor gene family were associated with sweet taste perception and the intake of sweet foods^(8–10)^. However, these variants suggested by candidate gene studies could not be replicated by subsequent genome-wide association study (GWAS) in both samples of European ancestry and Japanese, probably because the previous candidate gene studies did not consider population stratification^(11,12)^. In GWAS, rather than focusing on biological candidate genes, the genome is screened without any prior predilection for specific regions, genes, or variants thereof. Thus, GWAS have been characterised as ‘hypothesis-free’ approaches^(13)^. Recently, several GWAS on the dietary habits of the Japanese population have shown interesting pleiotropic effects of the single nucleotide polymorphism (SNP) rs671, which encodes aldehyde dehydrogenase 2 (*ALDH2*) genes, on dietary habits including foods and beverages. We and others found that an effect rs671 allele was inversely associated with fish consumption^(14,15)^, whereas it was directly associated with coffee consumption^(16)^. Matoba *et al.* showed that rs671 had no association with meat consumption, but they did not show whether any other genetic variants were significantly associated with meat consumption in Japanese^(17)^. The only report of a GWAS on dietary intake, including meat, in samples of European ancestry that we found was that by Niarchou *et al.*^(18)^. They identified twenty-nine independent SNPs associated with diet component 1, obtained by a principal component analysis (PCA) that represented a meat-related diet.

The purposes of this study were (1) to perform a GWAS on total meat consumption in a Japanese population and (2) to replicate the results of the GWAS on meat consumption in samples of European ancestry in a Japanese population.

## Methods

### Study population

This cross-sectional study was conducted with participants aged from 35 to 69 years as part of the Japan Multi-Institutional Collaborative Cohort (J-MICC) study that started in 2005 to investigate gene-environment interactions in lifestyle-related diseases. We used the data ver. 20180112. The 14 539 participants in the J-MICC study were recruited from twelve different areas throughout Japan between 2004 and 2014. Details of the J-MICC study were reported elsewhere^(14,16,19)^. Briefly, participants completed a questionnaire about lifestyle and medical information and gave a blood sample at the time of the baseline survey. The J-MICC study participants included community citizens, first-visit patients to a cancer hospital and health check examinees. All participants in this study gave written informed consent, and the study protocol was approved by the Research Ethics Committees of Aichi Cancer Center, Nagoya University Graduate School of Medicine, and the other institutions participating in the J-MICC study. The present study was conducted according to the principles expressed in the World Medical Association Declaration of Helsinki.

Of 14 539 participants, 448 were excluded based on the GWAS screening described in the ‘Genotyping and quality control filtering’ section. Of the remaining 14 091 participants, two withdrew from the study afterwards, three were outside of the study age range, seven had daily energy intake less than 500 kcal or greater than 5000 kcal and three with missing nutritional data were excluded. As a result, we analysed the data of 14 076 participants in the present study.

### Questionnaire and measurements

The questionnaire for the J-MICC studies included questions about medical history, height, weight, smoking and drinking habits, and dietary habits. The questionnaire was checked by experienced staff to confirm completeness and consistency. Height and weight measurements and blood sampling were conducted as part of a health check-up or for research purposes at the institutions participating in the J-MICC study^(19)^. Question items were collected using a scientifically validated self-administered questionnaire^(20–24)^. Body mass index (BMI) was calculated by dividing body weight in kilograms by the square of height in metres.

### Dietary assessment

We used a semi-quantitative food frequency questionnaire (FFQ) to estimate food intake that has been reported previously^(20–25)^. We chose twenty foods/food groups and beverages (shown as <number>) including <1> rice, <2> bread, <3> noodles, <4> potatoes, <5> soyabeans, <6> soyabean-paste, <7> green-yellow vegetables, <8> other vegetables, <9> fruit, <10> mushrooms, <11> seaweed, <12> fish and other seafood, <13> meat (chicken, beef or pork, liver, ham group [including sausage, salami and bacon]), <14> eggs, <15> milk, <16> oils, <17> confectionery, <18> green tea, <19> coffee and <20> alcoholic beverages. Food intake frequencies were classified into eight categories (never or seldom, one to three times per month, one to two times per week, three to four times per week, five to six times per week, once a day, twice a day and three or more times a day, which were converted into 0, 0⋅1, 0⋅2, 0⋅5, 0⋅8, 1, 2 and 3 before analysis). For each food category, the frequency was multiplied by the portion size, and the total intake amount was calculated. For the present study, total meat consumption was extracted. Energy intake by FFQs was estimated by using the Standard Tables of Food Composition in Japan, 5th edition^(26)^. Total alcohol intake was estimated as the sum of pure alcohol intake. The frequency of alcohol intake was obtained in six categories (never or seldom, one to three times per month, one to two times per week, three to four times per week, five to six times per week and every day). Total alcohol consumption (g/d) was estimated as the summed amount of pure alcohol consumption.

### Genotyping and quality control filtering

Buffy coat fractions and DNA were prepared from blood samples and stored at −80 °C at the central J-MICC Study office. DNA was extracted from all buffy coat fractions using a BioRobot M48 Workstation (Qiagen Group, Tokyo, Japan) at the central study office. For the samples from two areas (Fukuoka and Kyushu and the Okinawa Population Study [KOPS]), DNA was extracted locally from samples of whole blood using an automatic nucleic acid isolation system (NA-3000, Kurabo, Co., Ltd, Osaka, Japan). The 14 539 study participants from the thirteen areas of the J-MICC study were genotyped at the RIKEN Center for Integrative Medicine Sciences using a HumanOmniExpressExome-8v1.2 BeadChip array (Illumina Inc., San Diego, CA, USA). Twenty-six participants with inconsistent sex information between the questionnaire and the estimate from genotyping were excluded. The identity-by-descent method in the PLINK 1.9 software^(27,28)^ identified 388 close relationship pairs (pi-hat > 0⋅1875), and one sample from each pair of the 388 was excluded. PCA^(29)^ with a 1000 Genomes reference panel (phase 3)^(30)^ detected thirty-four participants whose estimated ancestries were outside the Japanese population^(31)^. These thirty-four participants were excluded. In the remaining 14 091 participants, SNPs with a genotype call rate of <0⋅98 and/or a Hardy–Weinberg equilibrium exact test *P*<1×10^−6^, a low minor allele frequency (MAF) < 0⋅01 or a departure from the allele frequency computed from the 1000 Genomes Phase 3 EAS samples were excluded. The quality control filtering resulted in 14 091 individuals and 574 423 SNPs.

### Genotype imputation

Genotype imputation was performed using SHAPEIT^(32)^ and Minimac3 softwares^(33)^ based on the 1000 Genomes Phase 3 all ancestries as a reference panel^(30)^. After genotype imputation, strict quality control filters were applied; namely, variants with an *R*^2^ < 0⋅3 were excluded, resulting in 12 617 547 variants. Finally, 4 112 564 variants with MAF < 0⋅01 in patients were removed, resulting in 8 503 383 variants for the analysis. We used the DosageConvertor software^(34)^ to convert dosage files in VCF formats from Minimac3 to PLINK formats.

### Power calculations to test for an association between total meat intake and SNPs

Statistical power to detect a true association was calculated by the method of Delongchamp *et al.*^(35)^, based on the number of participants and genetic data of the discovery phase J-MICC study. The required non-centrality parameter was obtained by the equation A4 listed in Appendix A by Visscher *et al.*^(36)^. When then the number of participants is 14 076, with fourteen covariates for adjustment, 0⋅8 for linkage disequilibrium (LD) *R*^2^, 0⋅2 for MAF, 0⋅02 for a squared standardised *β* estimate and 8 500 000 for variants analysed, the statistical power is calculated as 0⋅992 according to the method proposed by Delongchamp *et al.*^(35)^.

### Association analyses between genetic variants and total meat intake

Associations between all imputed variants and total meat intake were analysed by linear regression assuming the additive effects of the allele dosage on total meat intake per 1000 kcal energy intake (g/1000 kcal per d) adjusted for age, sex, and PCA components 1–10 using the PLINK 1.9 software^(27,28)^. We also performed a sex-stratified linear regression analysis, because there were significant differences in dietary intake between men and women. Furthermore, we performed logistic analysis by dichotomising meat intake per 1000 kcal at the sex-specific median in low *v.* high adjusted for age, sex and PCA components 1–10, because our use of semi-quantitative FFQ might not be suited to use dietary intake as an absolute continuous variable. We also performed a sex-stratified logistic analysis. Variants achieving genome-wide significance (*P* < 5 × 10^−8^) were considered as total meat intake-associated variants. An R package for creating a quantile–quantile (Q–Q) plot, GWAS tools, was used^(37)^. For scatter plots of *P*-values derived from genome-wide scan results for total meat intake, the qqman software was used^(38)^.

For a sensitivity analysis, associations between all imputed variants and beef and pork intake, rather than total meat intake, were analysed adjusted for the same variables as above.

In addition, replication analysis on meat intake per 1000 kcal adjusted for age, sex and PCA components 1–10 using the J-MICC samples for twenty-nine SNPs that were previously reported to be associated with total meat intake^(18)^ was performed.

Student's *t*-tests were used to compare means between men and women.

## Results

### Baseline characteristics

Baseline characteristics of the total, male and female, participants are shown in Table 1. The mean age of the participants was 54⋅8  years, and the percentage of women was 55⋅0 %. The mean total meat intake was 37⋅9  g/d. The mean total energy intake (including that from alcohol) was 1768  kcal/d, and the mean total meat intake per 1000 kcal energy intake was 22⋅0  (g/1000 kcal per d). The means for protein, fat, carbohydrate (% of total energy) and alcohol intake (g/d) were 13⋅7 , 25⋅7 , 60⋅6% and 9⋅4 g/d, respectively. The mean BMI was 23⋅1 kg/m^2^. The mean age, total energy intake, percentage of carbohydrate intake, alcohol intake and BMI were significantly larger in men than in women. The mean total meat intake, mean total meat intake per 1000 kcal, percentage of protein and percentage of fat intake were significantly smaller in men than in women. The median total meat intake per 1000 kcal for men and women were 15⋅36 and 22⋅65 g/1000 kcal per d, respectively.

**Table 1.** Background characteristics of the study participants (J-MICC)

	Total	Men	Women	*P*
*N*	14 076	6332	7744	
Age (years)	54⋅8 (9⋅4)	55⋅4 (9⋅3)	54⋅3 (9⋅4)	<0⋅001
Meat (g/d)	37⋅8 (22⋅4)	35⋅9 (21⋅8)	39⋅4 (22⋅7)	<0⋅001
Meat per 1000 kcal (g/1000 kcal per d)	22⋅0 (12⋅9)	17⋅9 (10⋅4)	25⋅3 (13⋅7)	<0⋅001
Energy (kcal/d)	1768 (411)	2026 ( 413)	1556 (263)	<0⋅001
PerP (%)	13⋅7 (1⋅7)	13⋅3 (1⋅7)	14⋅1 (1⋅6)	<0⋅001
PerF (%)	25⋅7 (5⋅9)	22⋅9 (5⋅4)	27⋅9 (5⋅3)	<0⋅001
PerC (%)	60⋅6 (7⋅0)	63⋅8 (6⋅5)	58⋅0 (6⋅2)	<0⋅001
Alcohol (g/d)	9⋅4 (16⋅3)	17⋅3 ( 20⋅2)	2⋅9 (7⋅6)	<0⋅001
BMI (kg/m^2^)	23⋅1 (3⋅3)	23⋅8 ( 3⋅1)	22⋅5 (3⋅3)	<0⋅001

BMI, body mass index; J-MICC, Japan Multi-Institutional Collaborative Cohort; PerP, percentage energy from protein; PerF, percentage energy from fat; PerC, percentage energy from carbohydrate.

Values are shown as the mean  (sd). *P*-values are by Student's *t*-tests.

### Association analyses between total meat intake and genetic variants

**Fig. 1.**
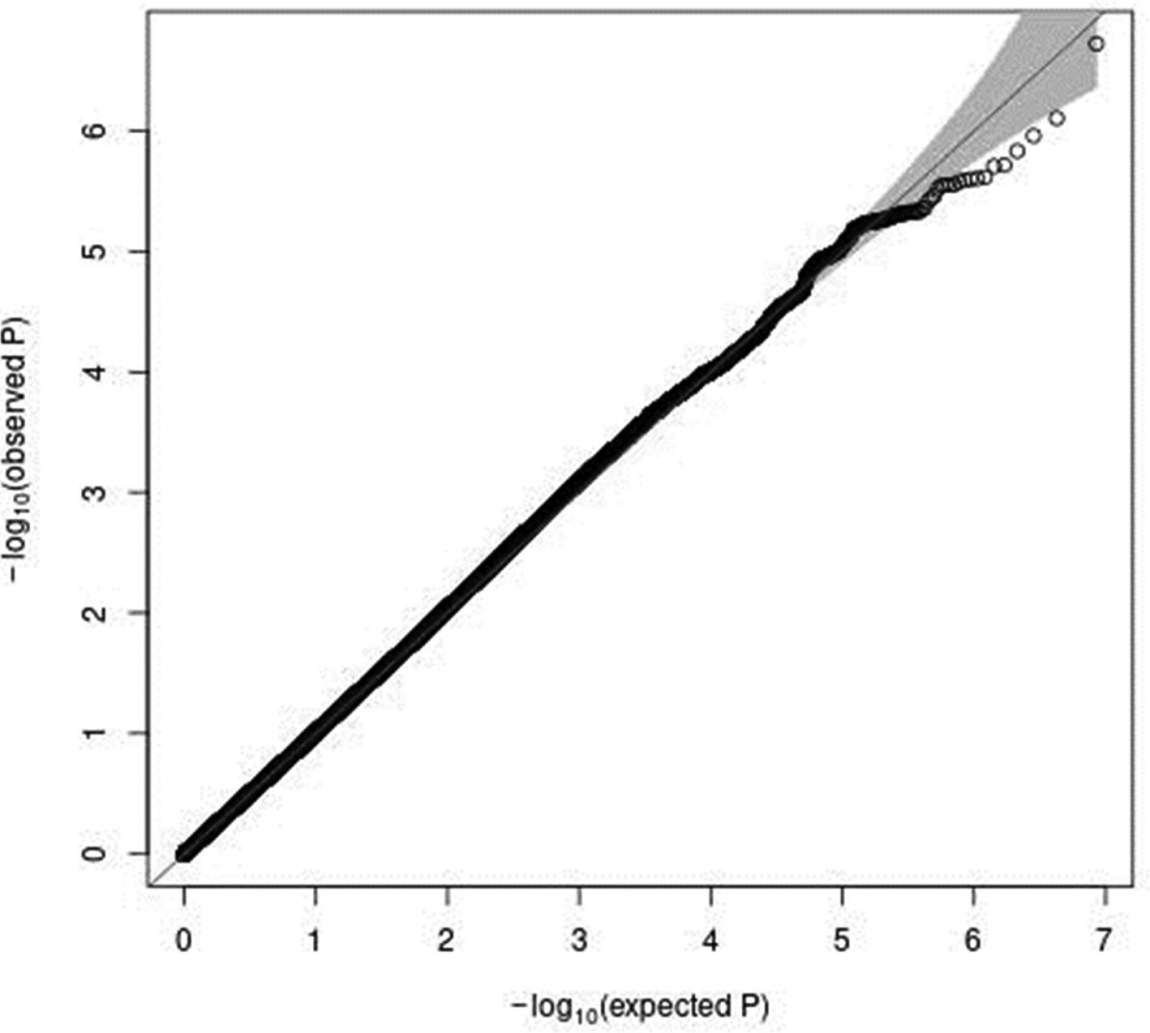
A Q–Q plot (black) for the GWAS of meat intake (g/1000 kcal per d). The *x*-axis shows the expected −log_10_ *P*-values under the null hypothesis. The *y*-axis expresses the observed −log_10_ *P*-values obtained by a linear regression model using PLINK^(27,28)^. The line represents *y* = *x*, which corresponds to the null hypothesis. The grey shaded area expresses the 95 % CI of the null hypothesis. The inflation factor (*λ*) is the median of the observed test statistics divided by the median of the expected test statistics (*λ* = 1.0117 [95% CI 1.0010–1.0131]). An R package for creating the Q–Q plot, GWAS tools, was used^(37)^. Chromosomal position (GRCh37/hg19).

**Fig. 2.**
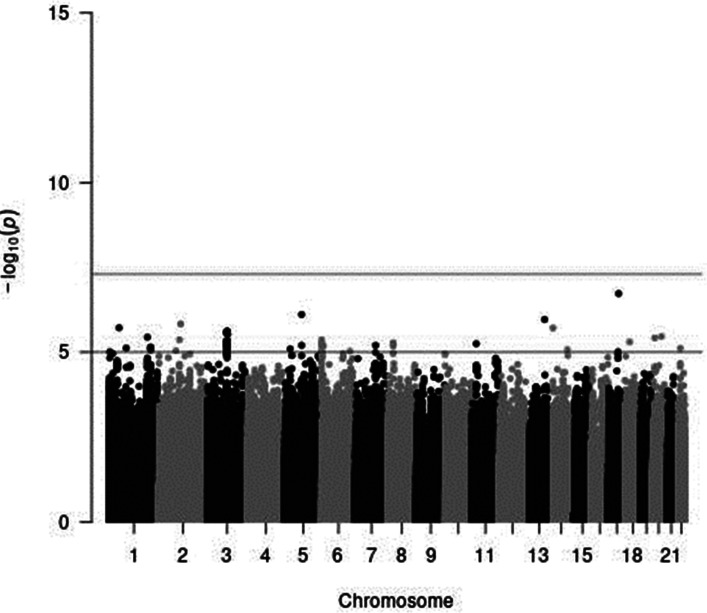
A Manhattan plot of the results from the GWAS of meat intake (g/1000 kcal per d). The *x*-axis indicates chromosomal positions, and the *y*-axis represents −log_10_ *P*-values obtained by linear model association analysis. The software qqman was used^(38)^. Chromosomal position (GRCh37/hg19).

In genome-wide analyses among the 8 503 383 variants adjusted for age, sex and PCA components 1–10, no variant was associated with total meat intake per 1000 kcal energy with genome-wide significance (*P* < 5 × 10^−8^). The Q–Q plot of the observed *P*-values is shown in Fig. 1. The inflation factor of the genome-wide scan was 1⋅0117 (95 % CI 1⋅0010, 1⋅0131), indicating that the population structure was well adjusted. Fig. 2 shows a Manhattan plot of the results from the GWAS of meat intake (g/1000 kcal per d), which found none with genome-wide significance (*P* < 5 × 10^−8^).

A sensitivity analysis with outcome variables restricted to beef and pork consumption yielded similar results.

**Fig. 3.**
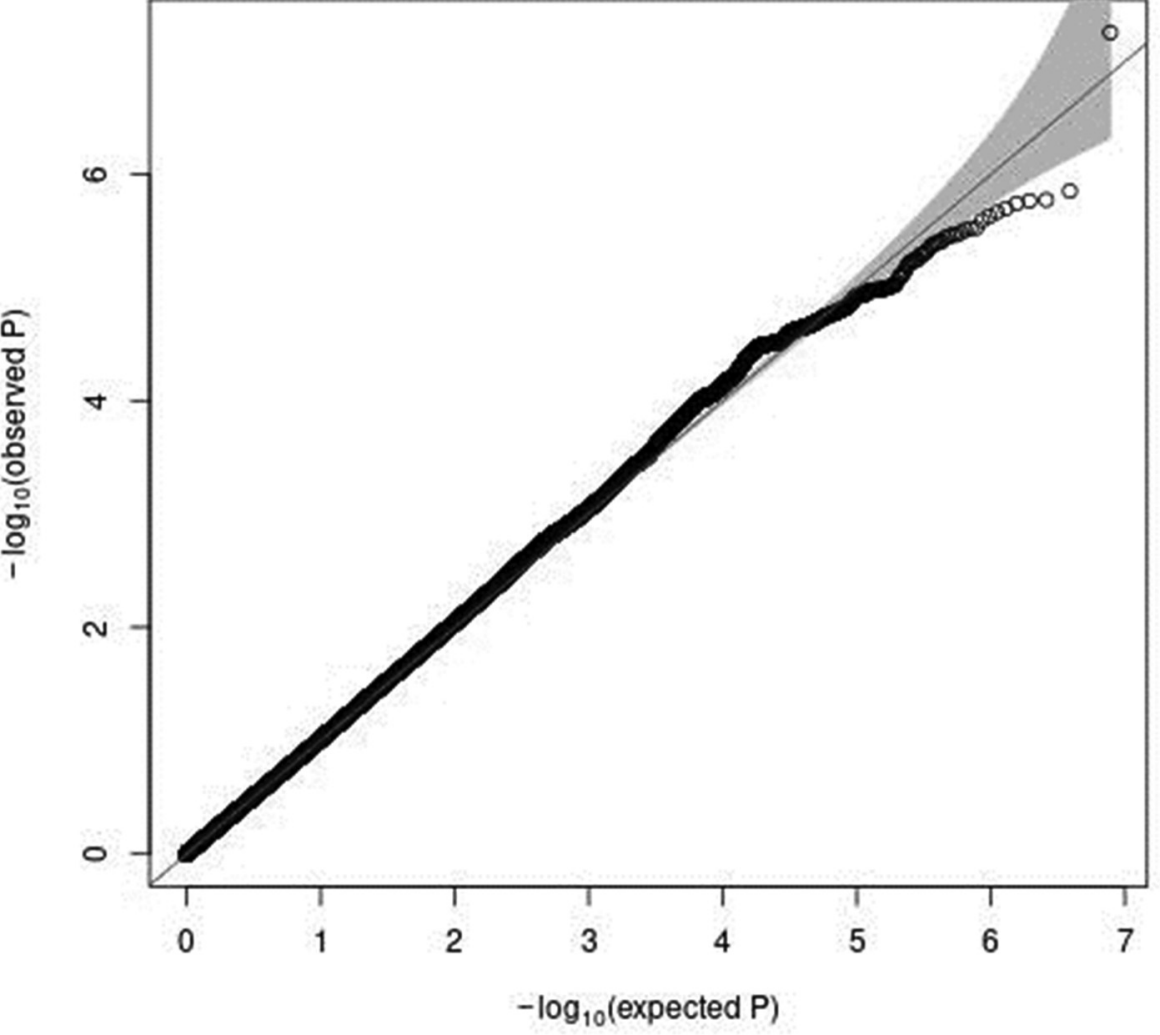
A Q–Q plot (black) for the sex-stratified GWAS of meat intake (g/1000 kcal per d) in women. The *x*-axis shows the expected −log_10_ *P*-values under the null hypothesis. The *y*-axis expresses the observed −log_10_ *P*-values obtained by a linear regression model using PLINK^(27,28)^. The line represents *y* = *x*, which corresponds to the null hypothesis. The grey shaded area expresses the 95 % CI of the null hypothesis. The inflation factor (*λ*) is the median of the observed test statistics divided by the median of the expected test statistics. An R package for creating the Q–Q plot, GWAS tools, was used^(37)^. Chromosomal position (GRCh37/hg19).

**Fig. 4.**
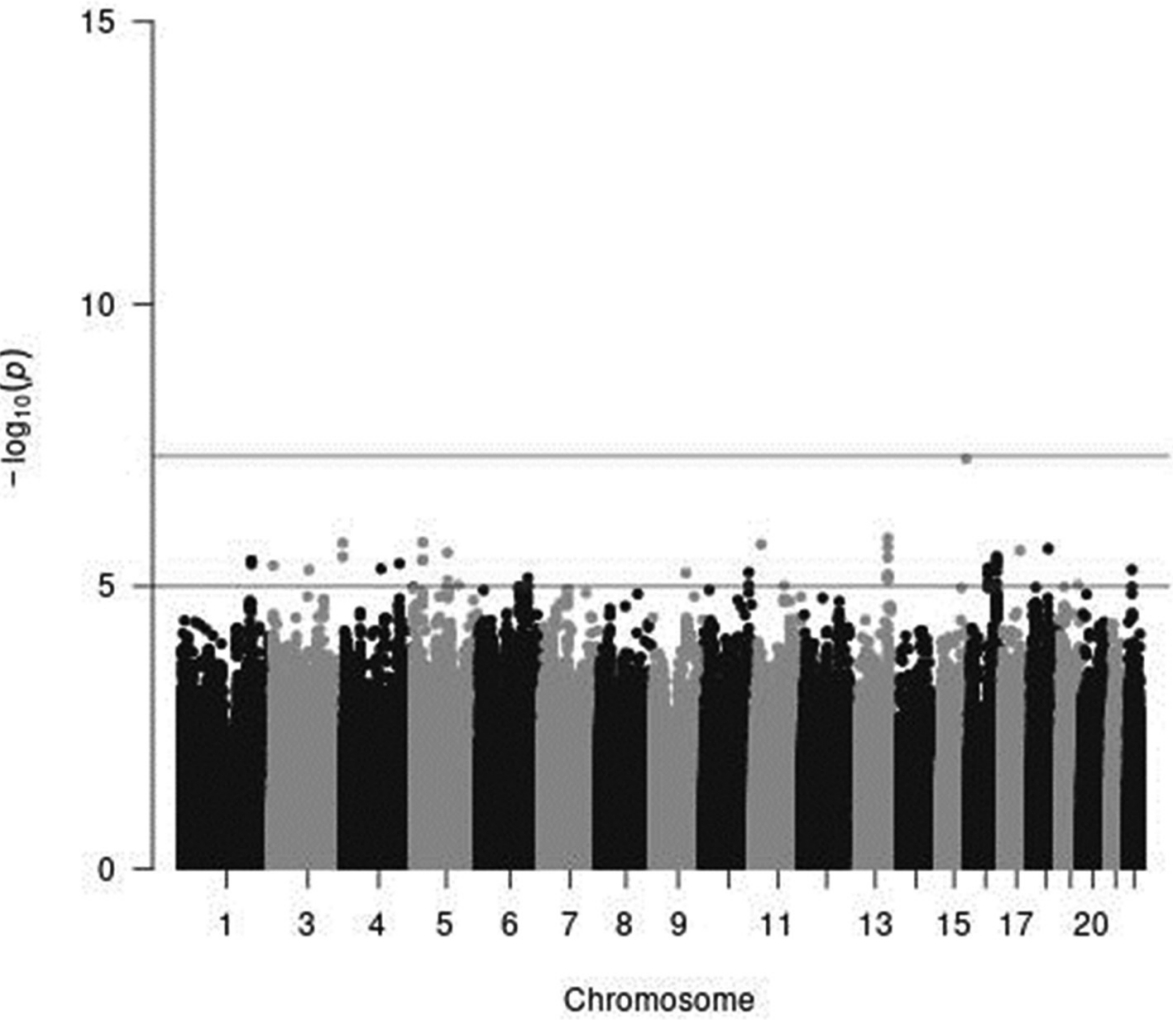
A Manhattan plot of the results from the GWAS of meat intake (g/1000 kcal per d) in women. The *x*-axis indicates chromosomal positions, and the *y*-axis represents −log_10_ *P*-values obtained by linear model association analysis. The software qqman was used^(38)^. Chromosomal position (GRCh37/hg19).

In a sex-stratified genome-wide linear regression analysis in men adjusted for age and PCA components 1–10, no variant was associated with total meat intake per 1000 kcal energy with genome-wide significance (the Q–Q plot of the observed *P*-values is shown in Supplementary Fig. S1 of Supplementary material, and a Manhattan plot of the results from the sex-stratified analysis in men is shown in Supplementary Fig. S2 of Supplementary material). However, in a sex-stratified genome-wide linear regression analysis in women, one variant, rs7166776 in 15q26.1, was marginally significantly associated with total meat intake per 1000 kcal energy (*P* = 5⋅54 × 10^−8^, Table 2). The Q–Q plot of the observed *P*-values is shown in Fig. 3, and a Manhattan plot in women is shown in Fig. 4.

**Table 2.** Result of a sex-stratified genome-wide linear regression analysis in women on total meat intake per 1000 kcal

SNP	Chr:BP	Genes	EA	NEA	EAFR	*β*	se	*P*
rs7166776	15:93865045	*LINC02207*	C	G	0⋅508	−1⋅291	0⋅237	5⋅54 × 10^−8^

SNP, single nucleotide polymorphism; Chr, chromosome, chromosomal position (GRCh37/hg19); BP, base pair positions; EA, effect allele; NEA, non-effect allele; EAFR, effect allele frequency; *β*, effect size; se, standard error of effect size.

Genome-wide analyses among the 8 503 383 variants adjusted for age, sex and PCA components 1–10, one variant, rs7166776 in 15q26.1, was marginally significantly associated with total meat intake per 1000 kcal energy.

In men and women combined logistic analysis by dichotomising meat intake per 1000 kcal at the sex-specific median adjusted for age, sex and PCA components 1–10, no variant was associated with low *v.* high total meat intake with genome-wide significance (the Q–Q plot of the observed *P*-values is shown in Supplementary Fig. S3 of Supplementary material, and a Manhattan plot is shown in Supplementary Fig. S4 of Supplementary material). Sex-stratified logistic analysis in men and women did not show any variant that was associated with low *v.* high total meat intake with genome-wide significance (a Manhattan plot in men is shown in Supplementary Fig. S5 of Supplementary material, and that in women is shown in Supplementary Fig. S6 of Supplementary material).

### Replication of previously reported SNPs

The results of a replication study in our J-MICC GWAS data with adjustment for age, sex, and PCA components 1–10 on the twenty-nine SNPs that were previously reported to be associated with diet component 1, obtained by a PCA, which represented a meat-related diet, are shown in Table 3. None of the SNPs reported were statistically significant (*P* < 0⋅05/29 = 0⋅0017) in Bonferroni correction.

**Table 3.** Replication analysis using the J-MICC samples for SNPs that were associated with meat intake in a previous study

SNP	Chr:BP	Genes	EA	NEA	EAFR	*β*	se	*P*
rs3101341	1:72747844	*NEGR1*	T	C	0⋅800	−0⋅068	0⋅183	0⋅709
rs66495454	1:72748567	*NEGR1*	GTCCT	G	0⋅234	0⋅240	0⋅177	0⋅174
rs506589	1:177894287	*SEC16B*	C	T	0⋅270	0⋅030	0⋅165	0⋅856
rs36016753	1:187269477	*-*	A	G	0⋅185	0⋅243	0⋅189	0⋅198
rs10900457	1:205146726	*CNTN2, RBBP5, etc.*	A	G	0⋅461	0⋅144	0⋅148	0⋅330
rs62106258	2:417167	*-*	NA	NA	NA	NA	NA	NA
rs6786550	3:62560523	*CADPS*	C	T	0⋅680	0⋅085	0⋅156	0⋅586
rs7644667	3:69040601	*EOGT, TMF1, etc.*	C	T	0⋅064	0⋅386	0⋅299	0⋅196
rs13340130	3:81790970	*GBE1*	T	A	0⋅528	−0⋅002	0⋅146	0⋅990
rs701760	4:113439212	*NEUROG2, LARP7*	G	C	0⋅320	0⋅371	0⋅157	0⋅018
rs300046	5:37081705	*NIPBL, C5orf42, etc.*	G	A	0⋅500	−0⋅163	0⋅152	0⋅282
rs10064431	5:92950673	*FAM172A*	C	T	0⋅614	0⋅273	0⋅150	0⋅068
rs9379831	6:26175852	*HIST1H2AC, HIST1H1E, etc.*	A	C	0⋅165	−0⋅051	0⋅235	0⋅829
rs806794	6:26200677	*HIST1H2AC, HIST1H1E, etc.*	G	A	0⋅839	−0⋅078	0⋅238	0⋅744
rs35797675	7:72878044	*FZD9, BAZ1B, etc.*	G	T	0⋅108	−0⋅490	0⋅240	0⋅041
rs4034907	7:135050259	*CNOT4, NUP205*	GATAA	G	0⋅759	0⋅128	0⋅173	0⋅459
rs10125463	9:15677925	*CCDC171*	T	A	0⋅223	0⋅074	0⋅175	0⋅674
rs6478868	9:131927092	*FAM73B, DOLPP1, etc.*	C	T	0⋅106	−0⋅034	0⋅237	0⋅885
rs1912286	10:87318888	*-*	A	G	0⋅493	0⋅074	0⋅147	0⋅614
rs3909727	11:126587382	*KIRREL3*	NA	NA	NA	NA	NA	NA
rs4759074	12:54664097	*CBX5, HNRNPA1, etc.*	T	C	0⋅632	0⋅312	0⋅150	0⋅038
rs12103229	16:74167594	*-*	A	C	0⋅360	−0⋅117	0⋅152	0⋅443
rs12232804	19:42677807	*ATP1A3, GRIK5, etc.*	T	C	0⋅054	0⋅599	0⋅326	0⋅066
rs429358	19:45411941	*PVRL2, TOMM40, etc.*	C	T	0⋅101	0⋅392	0⋅244	0⋅109
rs838144	19:49250239	*FUT2, MAMSTR, etc.*	T	C	0⋅980	−0⋅097	0⋅642	0⋅880
rs79564737	20:43408372	*RIMS4*	A	G	0⋅535	−0⋅100	0⋅146	0⋅494
rs136528	22:27245262	*-*	C	G	0⋅634	0⋅105	0⋅152	0⋅491
rs139911	22:40704052	*TNRC6B*	T	C	0⋅461	−0⋅249	0⋅146	0⋅087
rs202637	22:41853928	*ZC3H7B, TEF, etc.*	G	A	0⋅954	0⋅312	0⋅349	0⋅371

SNP, single nucleotide polymorphism; Chr, chromosome, chromosomal position (GRCh37/hg19); EA, effect allele; NEA, non-effect allele; EAF, effect allele frequency; *β*, effect size; se, standard error of effect size. NA, not available in the J-MICC data – indicates no genes hit on that SNP.

We carried out a replication study on the twenty-nine identified SNPs associated with meat intake of European participants in the study by Niarchou *et al.*^(18)^.

## Discussion

In our previous GWAS on food consumption using the same dataset and a similar method, we found that an effect rs671 allele was inversely associated with fish consumption^(14)^, whereas it was directly associated with coffee consumption^(16)^. We also found one SNP in the 14q11.2 locus that was significantly associated with the Japanese food score^(39)^. However, in this study based on 14 076 Japanese, we found no significant association between tested variants and total meat per 1000 kcal energy intake, or beef and pork consumption, in a Japanese population. In a sex-stratified genome-wide linear regression analysis in women, one variant, rs7166776 in 15q26.1, was marginally significantly associated with total meat intake per 1000 kcal energy (*P* = 5⋅54 × 10^−8^). The rs7166776 SNP is an intron variant of gene LOC105370982, which is classified as a non-coding RNA, and no disorders were found for LOC105370982 gene. The sex-stratified logistic analysis in women in the present study could not replicate the association between rs7166776 and meat consumption. Thus, the finding in the stratified linear regression analysis in women is considered as a chance finding. Additionally, twenty-nine SNPs that previously reported in the different ethnicity was not replicated in the present study. Thus, these missing significant variants in the association tests may suggest the relatively small effect of the genetic factor for meat consumption in Japanese populations.

We have recently seen an increasing number of results on the dietary habits of the Japanese population. Most of the variants found were the SNP rs671, which encodes *ALDH2*, or other variants that have a high LD with rs671. For instance, an effect rs671 allele was inversely associated with fish consumption^(14)^, whereas it was directly associated with coffee consumption^(16)^. Furthermore, Matoba *et al.* in the BioBank Japan Project (BBJ) showed that an effect allele was inversely associated with natto and tofu consumption, and it was directly associated with green tea, milk and yogurt consumption. They also showed that an effect allele had a neutral association with vegetable and meat consumption^(17)^. Since BBJ is a hospital-based cohort that includes individuals affected with some of the target diseases, possible differences in dietary habits between pre-diagnosed and diseased individuals could have affected the results. Our present results from previous studies on coffee and fish consumption and the present results on meat consumption confirmed that the findings of the study by Matoba *et al.*^(17)^ in a hospital-based cohort held in a healthy population.

The reason rs671 has pleiotropic effects on food and beverage consumption in Japanese participants is not clear. Those who cannot tolerate alcoholic beverages may drink coffee, green tea and milk instead. Acetaldehyde is contained in fish and gives many foods a pleasant aroma^(40)^. Natto contains trace concentrations of acetaldehyde and some detectable concentrations of ethanol^(41)^. Acetaldehyde in fish and ethanol in natto may produce some unpleasant taste or smell in those with a defective *ALDH2* genotype, and thus they eat smaller amounts of fish and natto. One reason we did not find any effect of rs671 on meat consumption may be meat may not contain any substance that causes unpleasant taste or smell in those with a defective *ALDH2* genotype. Another aspect that may be related to why we did not find any genetic variant associated with meat consumption in a Japanese population is that the development of genetic interaction with some foods needs some extensive duration of exposure of some foods. With the arrival of Buddhism in the 6th century, Japanese people stopped eating meat until the late 19th century Meiji Era. But even today, the amount of meat consumption is far less than that in Western countries^(7)^. This lack of exposure to meat-eating for a long duration might have failed in the development of gene–meat-eating interaction.

This study has several limitations. We did not perform a replication study in a different Japanese population, because the present results were negative from a GWAS point of view. A replication study in a European population, however, would probably yield quite different results from those we found in the present study, since *ALDH2* polymorphism is restricted in Eastern Asian populations. Second, although we used a semi-quantitative FFQ to estimate food intake as reported previously^(19–24)^, the number of meat foods included in the FFQ was small. Furthermore, the use of semi-quantitative FFQ is not best suited to use dietary intake as an absolute continuous variable, because semi-quantitative FFQ, in general, does not reflect portion sizes accurately and relies solely on self-report. To compensate for shortcomings, we performed a logistic analysis by dichotomising meat intake in low *v.* high at sex-specific medians.

In conclusion, we found that no genetic variants, including rs671, were associated with total meat or beef and pork consumption; therefore, meat consumption was not influenced by genetic factors in a Japanese population.

## Supplementary material

For supplementary material accompanying this paper visit http://dx.doi.org/10.1017/jns.2021.49. click here to view supplementary material
