# Supplementary material for: A genome-wide association study on meat consumption in a Japanese population: the Japan Multi-Institutional Collaborative Cohort study
Source: J Nutr Sci. 2021 Oct 11;10:e61. doi: 10.1017/jns.2021.49 (PMC8532070; doi:10.1017/jns.2021.49)
Supplement: Supplementary file 1 [file S2048679021000495sup001.pdf]

Supplementary Figures

Figure S1

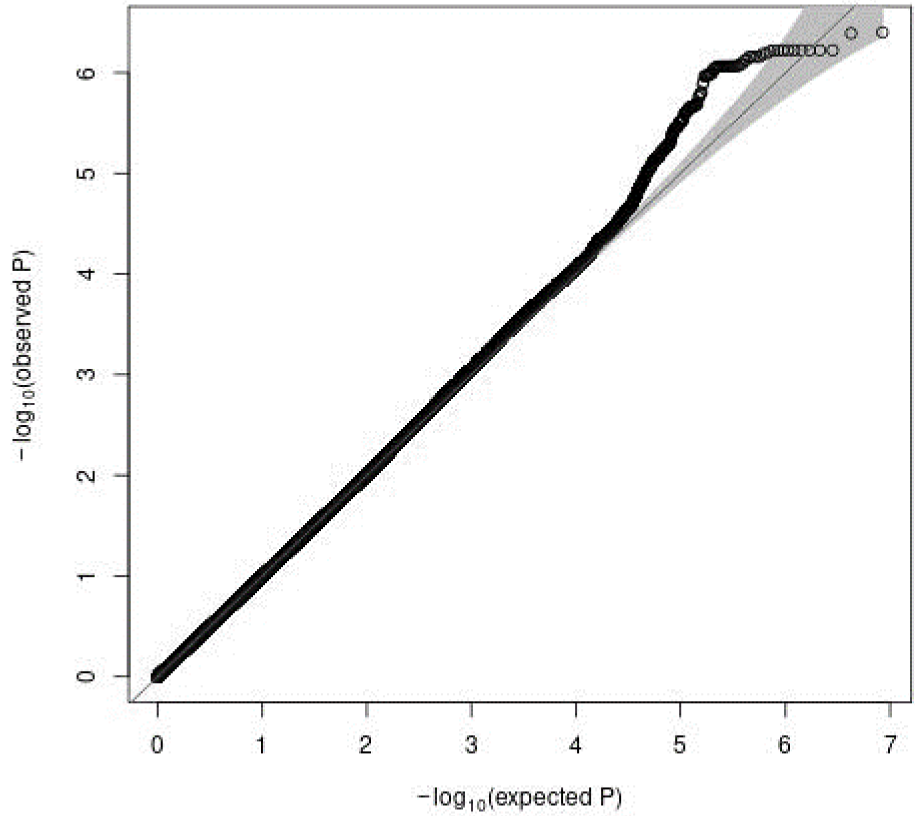

A quantile-quantile plot (black) for the sex-stratified GWAS of meat intake (g/1000 kcal/d) in men. The x-axis shows the expected -log10 P-values under the null hypothesis. The y-axis expresses the observed -log10 P-values obtained by a linear regression model using PLINK [27, 28]. The line represents y = x, which corresponds to the null hypothesis. The gray shaded area expresses the 95% confidence interval of the null hypothesis. The inflation factor (λ) is the median of the observed test statistics divided by the median of the expected test statistics. An R package for creating the Q-Q plot, GWASTools was used [37]. Chromosomal position (GRCh37/hg19)

Figure S2

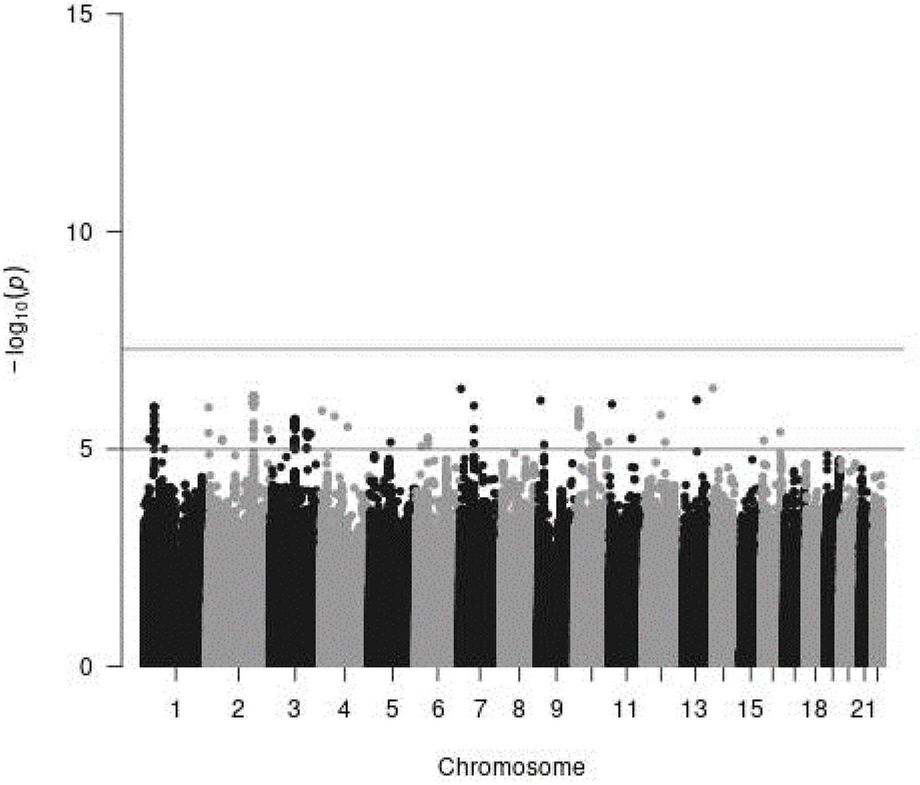

A Manhattan plot of the results from the sex-stratified GWAS of meat intake (g/1000 kcal/d) in men. The x-axis indicates chromosomal positions and the y-axis represents –log10 P-values obtained by linear model association analysis. The software qqman was used [38]. Chromosomal position (GRCh37/hg19).

Figure S3

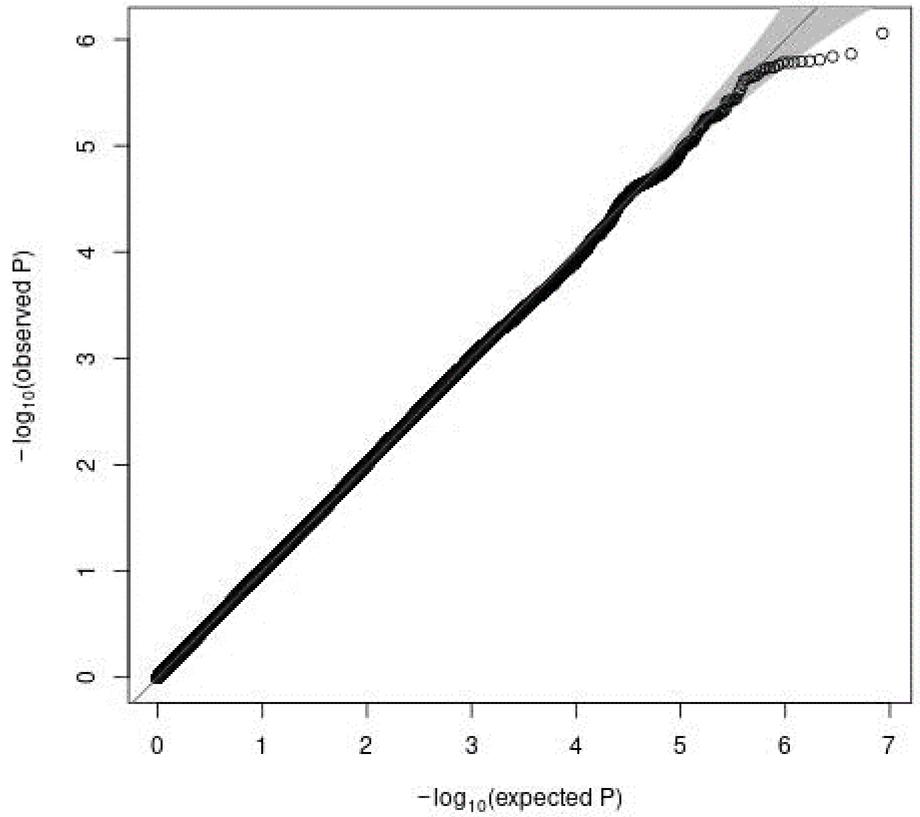

A quantile-quantile plot (black) for the GWAS by a logistic analysis of meat intake (high vs low). The x-axis shows the expected -log10 P-values under the null hypothesis. The y-axis expresses the observed -log10 P-values obtained by a linear regression model using PLINK [27, 28]. The line represents y = x, which corresponds to the null hypothesis. The gray shaded area expresses the 95% confidence interval of the null hypothesis. The inflation factor (λ) is the median of the observed test statistics divided by the median of the expected test statistics. An R package for creating the Q-Q plot, GWASTools was used [37]. Chromosomal position (GRCh37/hg19)

Figure S4

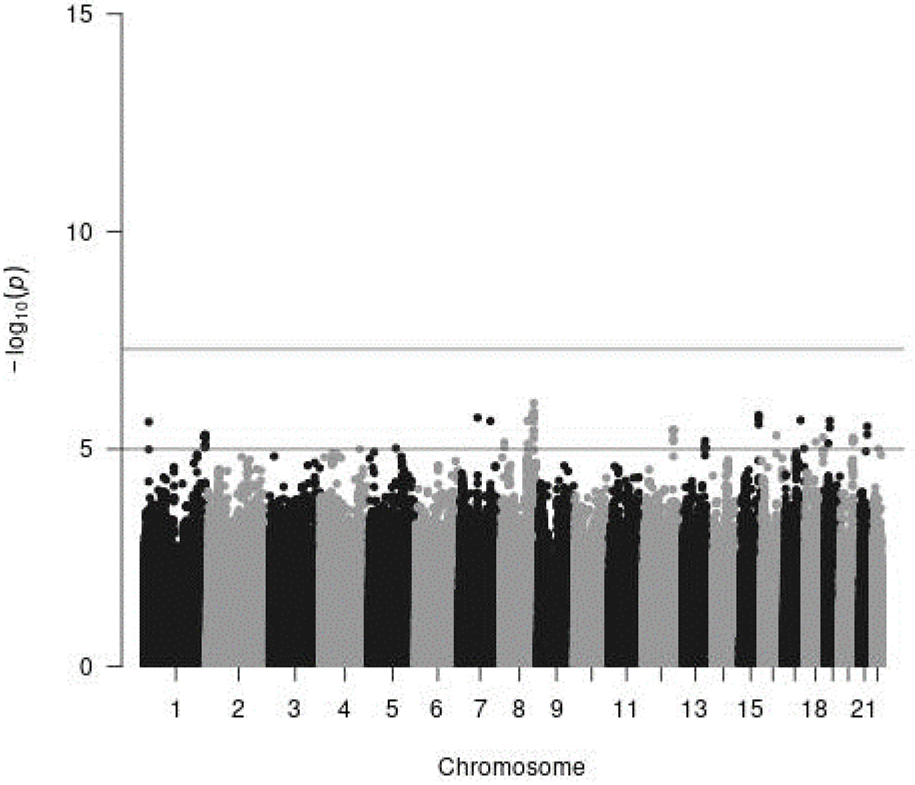

A Manhattan plot of the results from the GWAS by a logistic analysis of meat intake (high vs low). The x-axis indicates chromosomal positions and the y-axis represents –log10 P-values obtained by linear model association analysis. The software qqman was used [38]. Chromosomal position (GRCh37/hg19).

Figure S5

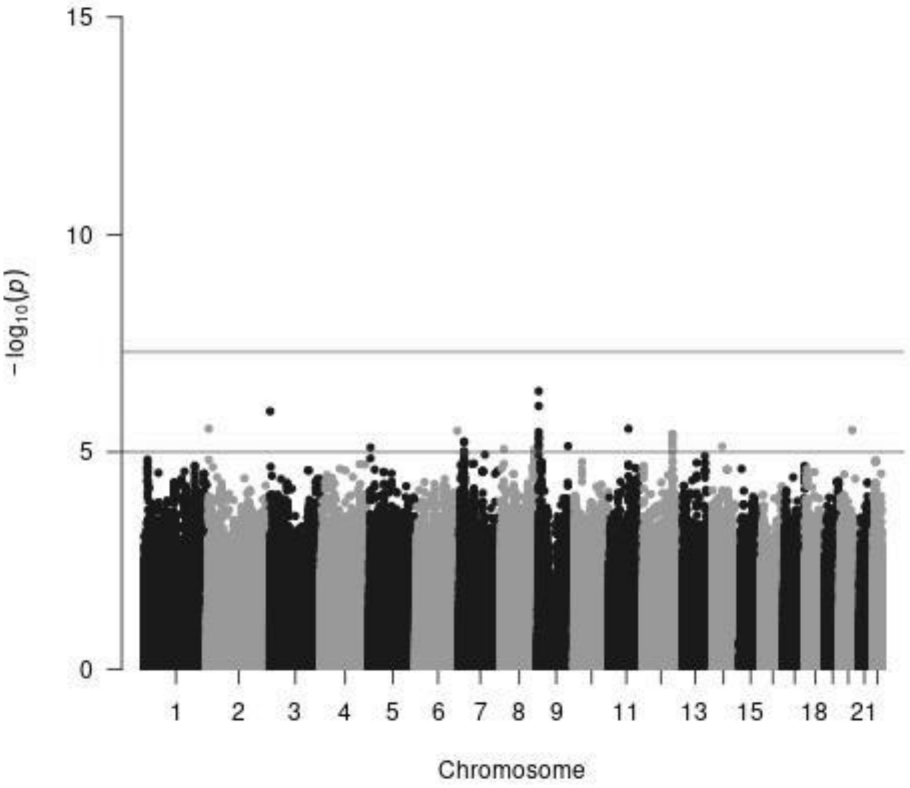

A Manhattan plot of the results from the GWAS by sex-stratified logistic analysis of meat intake (high vs low) in men. The x-axis indicates chromosomal positions and the y-axis represents –log10 P-values obtained by linear model association analysis. The software qqman was used [38]. Chromosomal position (GRCh37/hg19).

Figure S6

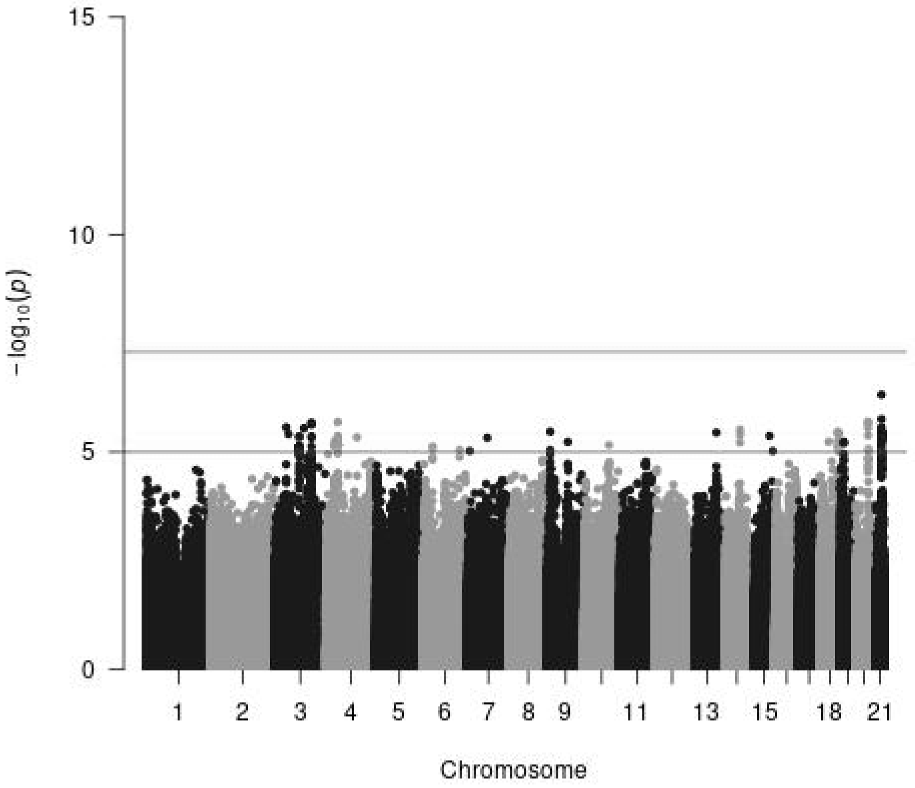

A Manhattan plot of the results from the GWAS by sex-stratified logistic analysis of meat intake (high vs low) in women. The x-axis indicates chromosomal positions and the y-axis represents –log10 P-values obtained by linear model association analysis. The software qqman was used [(38)]. Chromosomal position (GRCh37/hg19).
